# Good Statistical Monitoring: A Flexible Open-Source Tool to Detect Risks in Clinical Trials

**DOI:** 10.1007/s43441-024-00651-4

**Published:** 2024-05-09

**Authors:** George Wu, Spencer Childress, Zhongkai Wang, Matt Roumaya, Colleen McLaughlin Stern, Chelsea Dickens, Jeremy Wildfire

**Affiliations:** 1 Gilead Sciences Inc., 333 Lakeside Dr, Foster City, CA 94404 USA; 2 Atorus Research, Newtown Square, Harrisburg, PA USA

**Keywords:** Statistical monitoring, Risk-based quality management, Risk-based monitoring, Interactive graphics, R

## Abstract

**Background:**

Risk-based quality management is a regulatory-recommended approach to manage risk in a clinical trial. A key element of this strategy is to conduct risk-based monitoring to detect potential risks to critical data and processes earlier. However, there are limited publicly available tools to perform the analytics required for this purpose. Good Statistical Monitoring is a new open-source solution developed to help address this need.

**Methods:**

A team of statisticians, data scientists, clinicians, data managers, clinical operations, regulatory, and quality compliance staff collaborated to design Good Statistical Monitoring, an R package, to flexibly and efficiently implement end-to-end analyses of key risks. The package currently supports the mapping of clinical trial data from a variety of formats, evaluation of 12 key risk indicators, interactive visualization of analysis results, and creation of standardized reports.

**Results:**

The Good Statistical Monitoring package is freely available on GitHub and empowers clinical study teams to proactively monitor key risks. It employs a modular workflow to perform risk assessments that can be customized by replacing any workflow component with a study-specific alternative. Results can be exported to other clinical systems or can be viewed as an interactive report to facilitate follow-up risk mitigation. Rigorous testing and qualification are performed as part of each release to ensure package quality.

**Conclusions:**

Good Statistical Monitoring is an open-source solution designed to enable clinical study teams to implement statistical monitoring of critical risks, as part of a comprehensive risk-based quality management strategy.

## Introduction

Clinical trials aim to evaluate the safety and efficacy of promising therapeutic candidates, while protecting patients’ welfare and rights. To reliably achieve this objective, it is essential that both critical data and processes are high quality. Using traditional monitoring approaches like 100% source data verification and frequent site visits have been shown to be less efficient than a risk-focused strategy [1–3]. Regulatory authorities recommend risk-based monitoring (RBM) as a superior alternative, given it is a more adaptive and targeted approach to identify, assess, and mitigate study risks [4–6].

RBM is defined as five functional components - key risk indictors (KRIs), centralized monitoring, off-site/remote-site monitoring, reduced source data review, and reduced source data verification – and is part of a broader risk-based quality management framework, that also includes initial cross-functional risk assessment, ongoing cross-functional risk assessment, and quality tolerance limits [7]. The components collectively enhance the effectiveness of monitoring with proven benefits to trial quality, efficiency, patient safety, and overall value [8, 9]. Despite the advantages offered by RBM, adoption has been slower for risk detection components (32–35%) compared to risk assessment components (78–80%), even in the face of the increased need for remote risk detection through the recent COVID pandemic [10].

One possible driver for slower adoption is the lack of effective, easy-to-use, and inexpensive tools to properly perform risk detection compared to risk assessment. Recent reviews have found a breadth of tools available to assess potential risks to a trial at the start-up stage, but only limited information on how to develop or implement published methods for detecting study risk as the trial is ongoing [11–13]. In contrast, commercial and home-grown CRO solutions tend to be more sophisticated and include technical support, but are substantially more expensive to implement [14]. Given the proprietary nature of these systems, it is often difficult to share analysis findings and details of how the underlying risk detection algorithms work. Unfortunately, this trade-off between quality and cost may leave trial sponsors in a tough spot, especially when there are limited trial resources to support RBM.

To address this gap, we would like to introduce a new open-source R package, Good Statistical Monitoring {gsm}, as a free, flexible, and reliable tool to perform risk detection for RBM. R was chosen as it is freely available and widely used by the clinical trial community. GSM provides a supportive end-to-end framework for risk detection from data ingestion, risk analysis, visualization to reporting. It includes a flexible mapping process that is capable of handling multiple data standards and leverages a modular workflow structure that can easily be adjusted for study-specific customizations. It is also thoroughly tested and qualified prior to each release.

## Methods

{gsm} was designed based on a series of extensive discussions with clinicians, statisticians, data scientists, data managers, clinical operations, regulatory, and quality compliance staff, including reviews of existing tools and literature. The goal was to create a scalable and customizable analytics engine that could support an end-to-end workflow for risk detection including data ingestion, analysis, visualization, and reporting. Technical details, vignettes and example reports can be found at: https://gilead-biostats.github.io/gsm/index.html.

Development and testing of the functions in {gsm} relied primarily on two repositories of anonymized clinical trial data: {clindata} and {safetyData}. {safetyData} is an R package that reformats PHUSE’s sample ADaM and SDTM trial datasets [15]. {clindata} is a repository of anonymized and simulated clinical trial datasets from a variety of different sources and data formats [16].

Statistical analysis of KRIs in {gsm} relies on defining a numerator and a denominator for each metric (Table 1). Then depending on whether the metric is a percentage or a rate, the user can select different statistical methods to be applied. The default method is to use a normal approximation for percentages and rates, with an adjustment for overdispersion, to calculate z-scores for flagging at-risk sites [17]. When *m* sites are in a trial, where *m* > 2, the adjusted z-score for a site $i$ can be defined as:

**Table 1:** Key risk indicators

Metric	Definition
AE reporting rate	# of adverse events / days in study
SAE reporting rate	# of serious adverse events / days in study
G3+ lab abnormality rate	# of grade 3 or higher abnormal lab samples / total lab samples
Non-important PD rate	# of non-important protocol deviations / days in study
Important PD rate	# of important protocol deviations / days in Study
Subject discontinuation	# of subjects discontinued study / total enrolled participants
Treatment discontinuation	# of subjects discontinued treatment / total enrolled participants
Query rate	# of queries / total data points
Query age	# of queries open > 30 days / total queries
Visit entry lag	# of forms entered > 10 days / total forms
Date change rate	# of fields with ≥ 1 change / total fields
Screen failure	# of screen failures / total screened participants

The definition of each key risk indicator includes a numerator divided by a denominator. This represents an initial list of key risk indicators available in the current release and will be further updated in future releases

$${z{\prime }}_{i}=\frac{{y}_{i}-{\theta }_{0}}{\sqrt{V{\prime }\left(Y|{\theta }_{0}\right)}}$$

where 𝑦_𝑖_ is the KRI metric for site *i*, 𝜃_0_ is the overall mean, and 𝑉′(𝑌|𝜃_0_) is the over-dispersion adjusted variance. The over-dispersion parameter $\varphi$ is calculated as the average of unadjusted squared z-scores: $\varphi =\frac{1}{m}\sum _{i=1}^{m}{z}_{i}^{2}$. For percentages, the over-dispersion adjusted variance is $V{\prime }\left(Y|{\theta }_{0}=p\right)=\varphi \frac{\widehat{p}\left(1-\widehat{p}\right)}{n_i}$, where $\widehat{p}$ is the observed overall proportion of events and $n_i$ is the total number of study participants at site *i*. For rates, the over-dispersion adjusted variance is $V{\prime }\left(Y|{\theta }_{0}=\lambda\right)=\varphi \frac{\widehat{\lambda }}{{T}_{i}}$, where $\widehat{\lambda }$ is the observed exposure-adjusted incidence rate, defined as the total number of events divided by the total study exposure time and ${T}_{i}$ is the total exposure time for participants at site $i$. Alternatively, users can choose to perform Fisher’s exact tests for percentages and Poisson regression analyses for rates. More details can be found at https://gilead-biostats.github.io/gsm/articles/KRI%20Method.html.

Visualizations are built with R and JavaScript to create custom plots to depict analysis results. Interactive reports are produced as HTML documents using R Markdown. A detailed qualification report is automatically generated for each release using a set of machine-readable specifications and test cases to evaluate the expected performance of critical functions.

## Results

**Fig. 1:**
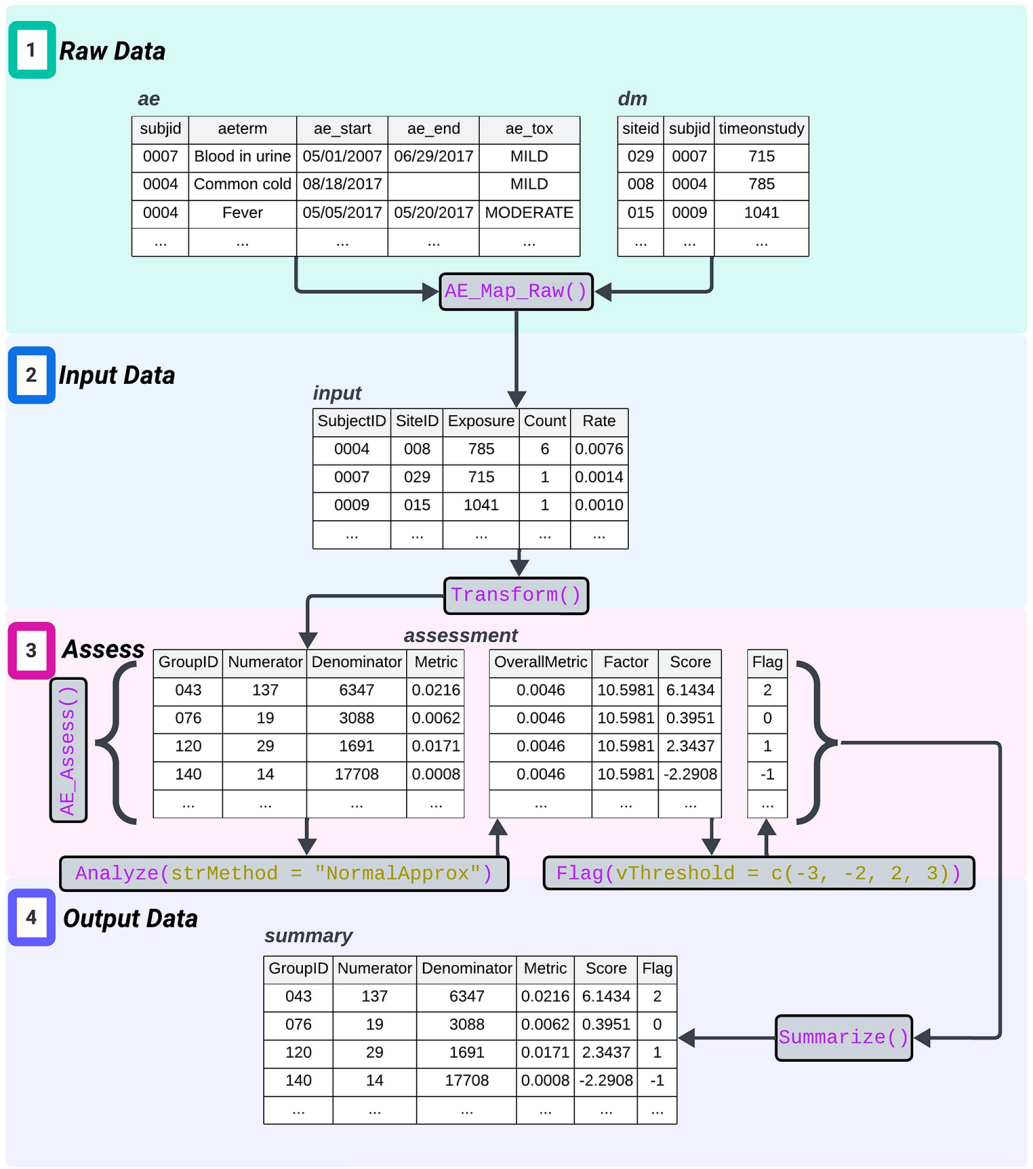
Assessment Model. This example illustrates the process to analyze the adverse event rate for each site, where datasets are shown as tables labled in italics, functions in purple and user-specified parameters in gold. First, the raw source data including adverse event data (*ae*) and subject data (*dm*) is mapped into the required input data format (*input*) containing the relevant information per subject, such as study exposure in days, number of adverse events, and the corresponding event rate. Next, the input data is transformed into an aggregate summary per site before being analyzed using the normal approximation method adjusting for over-dispersion. The resulting score is then flagged per the user-specified thresholds. Finally, the output is summarized for aggregation across all assessments performed for a study

**Fig. 2:**
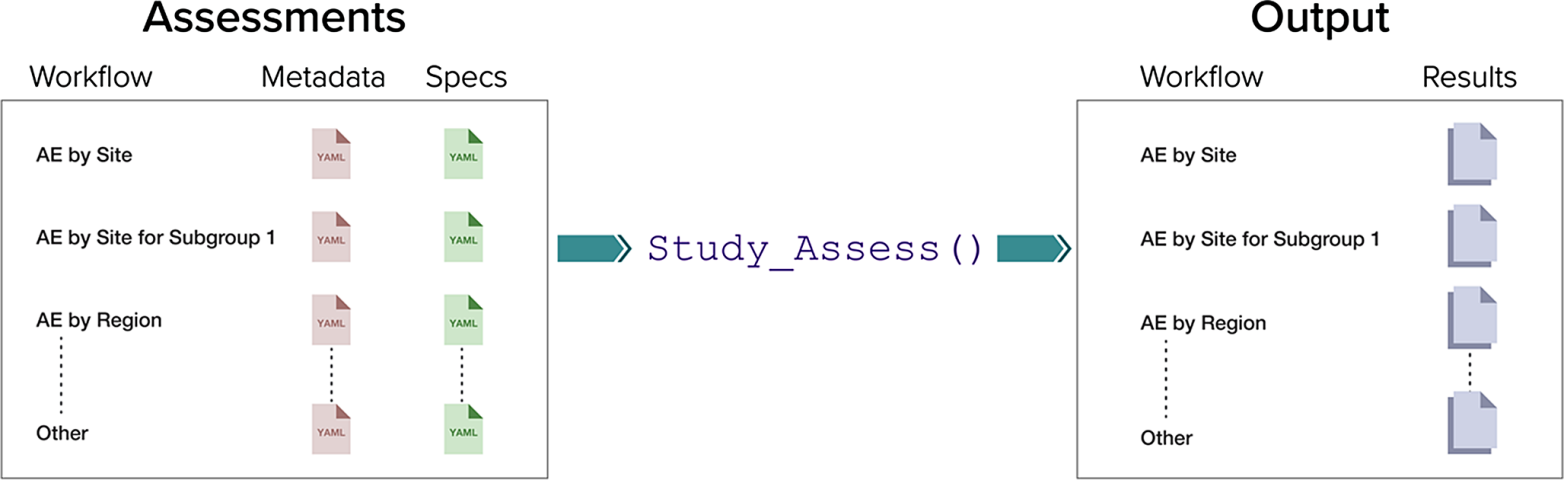
Workflow Structure. Users can stack workflows into a larger assessment object, where each workflow uses metadata and specifications inputted from separate YAML files to capture study-specific variable mappings, modifications to analyze by different groups (e.g., site, regions, etc.), filtering to exclude subjects from analyses, and other assessment settings. This object is then processed using the Study_Assess function producing an output object that contains results for each workflow

The analysis of each KRI in {gsm} is defined as an assessment following a standard model: data is first inputted at the trial participant level, transformed into a site-level summary, analyzed to generate test statistics and p-values, flagged to identify sites that cross user-specified thresholds, and then summarized (Fig. 1). Optional customizable mapping functions are provided to support conversion of trial data, from a variety of possible data sources and formats - ADaM, SDTM, raw, etc. - to the input data required for each assessment. Workflows expand upon assessments by adding more capabilities – support for country or region level analyses, analyses of data subsets, and automated data checking – and enable users to perform a set of workflows more easily and at scale through only one function (Fig. 2). An example of the benefit of being able to customize using workflows is a user can easily expand on an analysis of AE reporting rates for all enrolled patients, by adding filter functions to repeat the analysis in the same workflow focusing only on the subset of participants who were randomized and treated, or a subset of participants with a specific category of adverse events.

**Fig. 3:**
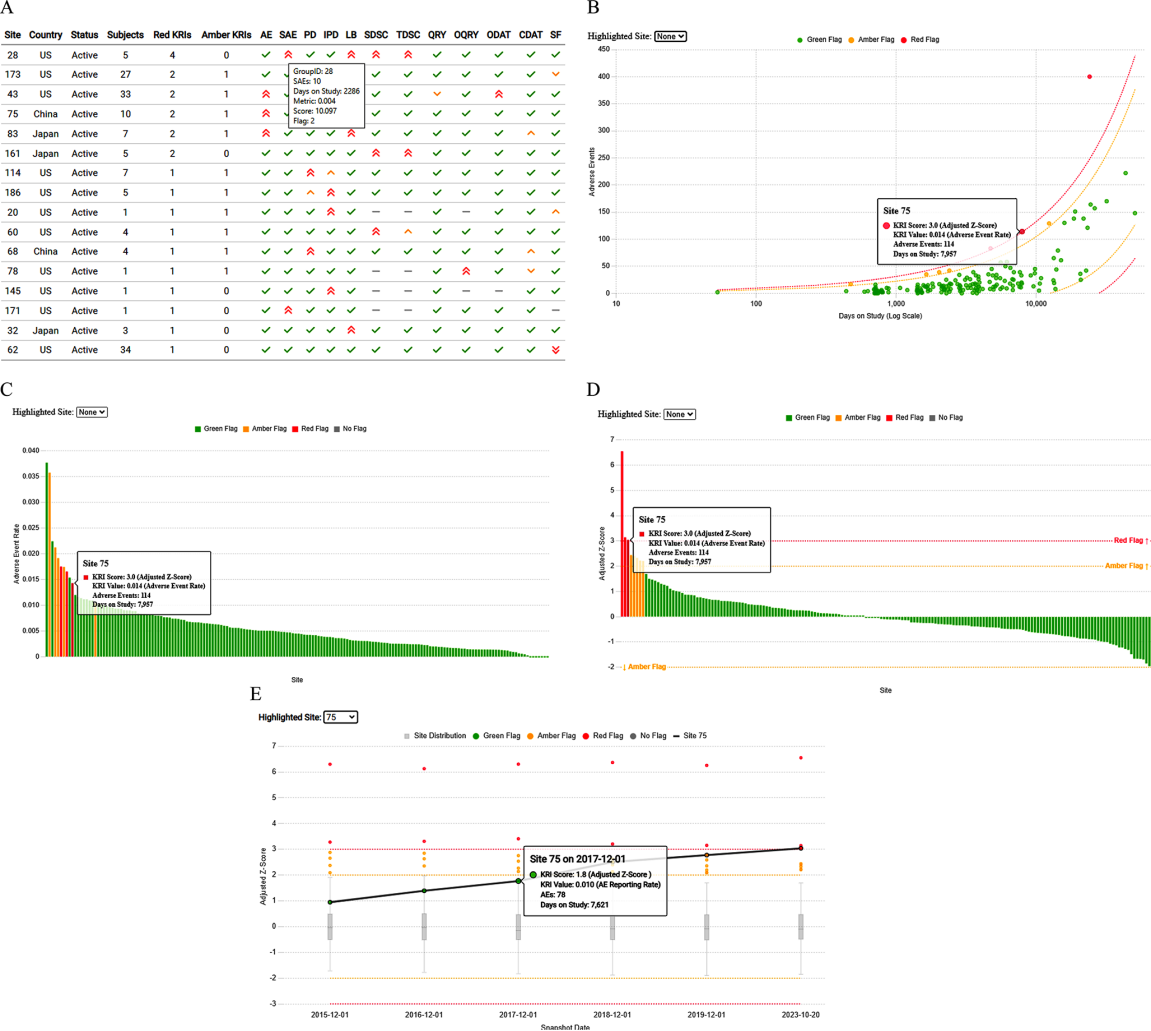
Visualizations. (**A**) Heatmap summarizing the findings for each KRI (column) by site (row), where the initial few columns provide information on the site identifier, country, status, number of subjects, total number of KRIs with red flags, and total # of KRIs with amber flags. Flags are determined by user-specified thresholds that indicate whether a finding should be flagged for further follow-up. Users can mouse-over specific study site and KRI findings to quickly understand more details about the results. Green check marks correspond to green flags, single amber arrows correspond to amber flags, and double red arrows correspond to red flags, where the direction of the arrow indicates whether the observed KRI rate for a given site is higher (up) or lower (down) than the overall KRI value across all sites in the study. (**B**) Plot of the number of adverse events (y-axis) by days on study on the log scale (x-axis) is shown, where each point is a site. Users can mouse-over individual points to see additional information such as the KRI score, KRI value, and underlying data used to derive the KRI for each site. Red, amber, and green dotted lines correspond to user-specified thresholds that determine whether sites should be flagged for further follow-up. (**C**) Barplot of adverse event rate sorted by sites with the highest to the lowest observed rates from left to right, where each bar is colored according to the flag for that site. (**D**) Barplot of adjusted z-scores calculated based on the KRI metric. Bars are sorted by sites with the highest to the lowest scores from left to right, where each bar is colored according to the flag for that site. Horizontal dotted lines indicate the user-specified thresholds for flagging. (**E**) Longitudinal plot of adjusted z-scores (y-axis) over repeated analyses at different snapshot dates (x-axis). Boxplots in gray summarize the distribution of z-scores at each snapshot, with flagged values in yellow and red highlighted along with the selected site of interest. Horizontal dotted lines represent the user-specified thresholds for flagging

{gsm} supports the creation of multiple interactive visualizations leading to a better understanding of analysis results. For individual assessments, results can be depicted as a scatter plot or bar plot on different scales (Fig. 3). For an overview of results, a site-by-assessment heatmap can be generated to highlight the commonly flagged KRIs across sites or the sites with the most flagged KRIs. For assessments of a given site over time, longitudinal plots can be created to show changes in results over multiple analyses. To easily capture and share the analysis results and visualizations, users can create a standard report with supportive trial information and the ability to search, filter, or examine specific data points of interest in more detail.

**Fig. 4:**
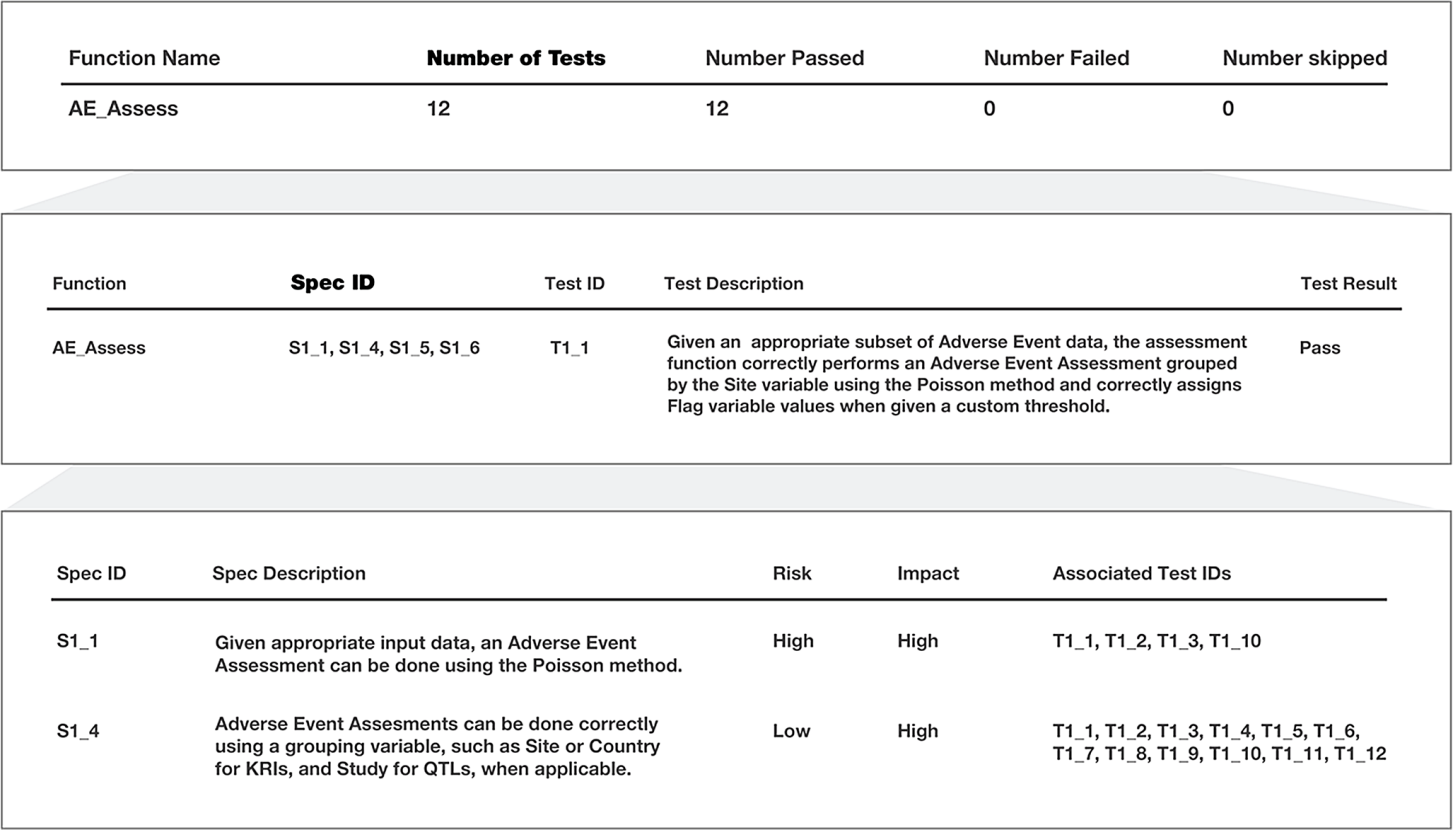
Qualification Report. Each function in the {gsm} package is tested prior to release, where each test is described in the report, and mapped to which specifications the tests are intended to evaluate. Each specification describes the particular outcome that is expected to be achieved, along with risk and impact scores (low, medium, high) depending on the level of risk and impact if the specification fails

The {gsm} R package has undergone extensive testing and qualification. As of v1.8.1, over 1,450 unit tests have been written with a 87.3% code coverage. Along with each release, a qualification report is automatically attached ensuring the package meets expected standards and requirements to detect study risks (Fig. 4). Qualification testing currently covers 24 core functions, evaluating 88 use cases across 171 total tests.

## Discussion

An effective RBM approach requires the ability to accurately detect study risks in a timely manner. {gsm} is a free open-source qualified solution developed for that purpose. It covers all the steps from data ingestion to reporting and allows R users to do so in a few lines of code. The modular structure of assessments and workflows facilitate study-specific customizations, and interactive visualizations allow users to better understand analysis results. Early efforts implementing {gsm} at Gilead have proven successful; we were able to detect similar risks as found by other proprietary systems, and more easily perform fit-for-purpose analyses for study-specific nuances across a diverse set of pilot studies.

Compared to alternative tools to detect risks as part of RBM, {gsm} offers a robust and effective solution for free. Among publicly available options, code to implement the proposed statistical methods may not exist, and if available, are usually provided in a piece-wise fashion or limited to a much narrower scope [18–20], making it difficult to detect all potential critical risks in a study, and impractical to systematically apply across a portfolio of studies. Commercial options typically offer a software-as-a-service approach [14] with more thorough and customizable analytics, but are substantially more expensive. Thus, {gsm} helps to fill an existing gap in risk detection tools and we hope will support increased adoption of RBM.

Future improvements planned for future releases, in order of prioritization, include expanding the number of KRIs that can be analyzed, supporting qualified QTL analyses, conducting unsupervised statistical monitoring, and incorporating more options for statistical testing. Current KRIs focus on critical areas related to study population, safety, deviations, and data quality, but do not yet cover other important areas such as primary and secondary endpoints, as this may require more complex study specific derivations and analyses. Although users can choose from more than one statistical method, some commonly used models like beta binomial models for binary outcomes [21], and linear mixed-effect models for continuous outcomes [22] have not been implemented. These methods may exhibit better performance in different situations; for example, the default method relying on normal approximation will tend to perform better when there are more sites, while an exact method may perform better when there are only a few sites. Further adding unsupervised approaches will allow users to agnostically survey the entirety of available trial data to find unknown risk signals. The {gsm} workflow can also easily be extended to perform QTL analyses, and experimental QTL functions, which need further refinement and validation, are being developed. Another interesting use case to explore is to use {gsm} to analyze real-world data to detect potential risks across regions, data sources, or other groupings. Adding these features will take time; fortunately {gsm} was purposely designed with a modular framework suited for quickly incorporating new improvements and releasing {gsm} as an open-source package will allow more R developers to contribute to its development.

Two of the primary drivers for releasing {gsm} as an open-source publicly available solution was to encourage collaboration with external partners, and benefit from the diverse experience of the broader R community. We believe this will result in much quicker integration of the latest statistical methods, expansion of the library of KRIs and QTLs that can be analyzed, creation of more visualizations, as well as faster discovery and resolution of bugs and pain points. A new PHUSE project called OpenRBQM was recently announced, which will combine an open-source RBQM Working group focused on information sharing with an RBQM Development Team that will co-develop new RBQM tools including {gsm}. We hope more and continued open collaboration will spur increased knowledge sharing on how to best perform risk based monitoring for the benefit of patients.

## Conclusion

{gsm} is an open-source qualified R package to ingest, analyze, identify, visualize, and report critical study risks with robust support for study-specific customizations. It is free for use under the Apache v2.0 license and has been successfully implemented on multiple clinical trials. We hope {gsm} will encourage more open collaboration to build better RBQM tools and achieve better outcomes for our trials and our patients. Full technical specifications, user guides, package details and more examples are available at https://gilead-biostats.github.io/gsm/.
